# Examining the impact of treatment guidelines on outpatient antibiotic prescription trends at a cancer center in Pakistan

**DOI:** 10.1017/ash.2025.2

**Published:** 2025-02-20

**Authors:** Salma Abbas, Seemal Aslam, Sara Batool, Mahnoor Zafar, Sadia Khaliq, Momal Fatima, Iraj Shehzad, Muhammad Arslan, Iqra Attiq, Muhammad Shehbaz, Anum Khan, Muhammad Ali Raza, Hamza Zulfiqar, Ahsan Mahmood, Faisal Sultan

**Affiliations:** 1 Department of Internal Medicine, Shaukat Khanum Memorial Cancer Hospital and Research Center, Lahore, Punjab, Pakistan; 2 Department of Acute Medicine and Infectious Diseases, Birmingham Heartlands Hospital, Birmingham, England

## Abstract

**Objective:**

To assess the impact of treatment guidelines on the trends of outpatient antibiotic prescription among pediatric and adult patients at a cancer center in Pakistan.

**Design:**

Retrospective observational study conducted between July 1^st^ 2018 and July 31^st^ 2023.

**Methods:**

We determined the indication for antibiotics and the frequency of guideline-discordant prescriptions for upper respiratory tract infection (URTI), lower respiratory tract infection (LRTI), urinary tract infection (UTI), and diarrhea. The χ^2^ test was used to assess the impact of treatment guidelines on antibiotics prescribed for these indications.

**Results:**

The top indications for antibiotic prescription were skin and skin structure infection (SSSI) (n = 5159; 21.5%), URTI (n = 2760; 11.5%) and UTI (n = 2686; 11.2%). Amoxicillin-clavulanate (n = 7964; 33.3%), was the most frequently prescribed antibiotic. A large proportion of antibiotic prescriptions for URTI, diarrhea, UTI, and LRTI were either inappropriate (n = 6695; 86.5%) or unnecessary (n = 5534; 71.5%). Results revealed a statistically significant decline in the proportion of inappropriate antibiotics for UTI (91.3% vs 84.0%; *P* ≤ .001) and diarrhea (92.6% vs 87.0%; *P* = .031) and unnecessary antibiotics for diarrhea (90.2% vs 83.2%; *P* = .016) with the introduction of treatment guidelines. We noted a higher proportion of unnecessary prescriptions for LRTI (41.7% vs 31.7%; *P* = .003) and inappropriate antibiotics for UTI (95.1% vs 87.4%; *P* = .011) for pediatric patients.

**Conclusion:**

Misuse of outpatient antibiotics is common. Diarrhea, URTI, UTI, and LRTI are high-priority conditions for outpatient oncology-focused prescriber education and stewardship interventions.

## Introduction

Antibiotics are among the most commonly prescribed medications in healthcare.^1^ Outpatient antibiotics account for over 60% of the total antibiotic expenditure in the United States of America (USA).^2^ About 20% of pediatric and 10% of adult outpatient visits result in antibiotic prescriptions.^3^ Azithromycin, amoxicillin, and ciprofloxacin are the most frequently prescribed outpatient antibiotics.^2,4–6^ Misuse of antibiotics is common and about 30 to 50% of all outpatient antibiotics are either inappropriate or unnecessary, indicating that the choice, dose, or duration of the anti-infective is incorrect or the antibiotic is prescribed without an appropriate indication.^3^ Common diagnoses for inappropriate antibiotic prescription include upper respiratory tract infection (URTI), presumed streptococcal pharyngitis, uncomplicated sinusitis, bronchitis, and certain ear infections.^2,3,7,8^ In Pakistan, granular data on outpatient antibiotic use are lacking and available figures are reliant on research studies limited in number and scale. Inappropriate antibiotic prescribing is rampant, facilitated by a culture of self-medication, over-the-counter dispensing of antibiotics without prescriptions, and poor access to healthcare and diagnostics.^9^ Amoxicillin, cefixime, and ciprofloxacin are among the most commonly prescribed antibiotics.^10,11^ Indications such as URTI and skin and skin structure infections (SSSIs) account for the majority of inappropriate prescriptions.^10^ Antibiotic prescribing varies across healthcare facilities with 12.1% office visits at tertiary care centers in major cities and 81.5% outpatient encounters in primary healthcare facilities resulting in antibiotic prescriptions.^10,12^

Injudicious antibiotic use fuels antimicrobial resistance and may lead to inflated healthcare costs, drug-related adverse events, and *Clostridioides difficile* infection (CDI).^2,3,8,13,14^ Deviation from recommended practices may be attributed to prescriber knowledge gaps, time constraints, patient expectations for antibiotic prescriptions, perceived association of antibiotic prescription with patient satisfaction, and diagnostic challenges in distinguishing viral infections from community-acquired pneumonia.^3,15–17^

Cancer patients represent a unique patient subset in that they receive immunosuppressive therapy along with antibiotics to prevent or treat infection.^18^ The threshold to prescribe is also lower in view of concerns regarding higher vulnerabilities in this population. In addition to expected complications such as CDI and adverse drug events, antibiotic misuse poses further challenges to cancer patients.^13,19^ The incidence of infections, particularly MDROs, and the associated mortality is higher compared to the general population.^18,20,21^ This may lead to delays in time-sensitive cancer treatments such as chemotherapy.^22^ Furthermore, a growing body of evidence suggests that alterations in microbiome diversity as a result of antimicrobial overuse may be associated with an increased risk of gastrointestinal graft versus host disease and poor response to immune checkpoint inhibitors.^20,23^ Antimicrobial stewardship (AS) efforts targeting cancer patients are likely to encounter resistance from oncology providers given the complexity of cases and the tendency to overtreat suspected or confirmed infections in immunocompromised hosts.^19,21,22^ This underscores the importance of establishing antimicrobial stewardship programs (ASP) at facilities providing cancer treatment. Oncology-focused AS strategies must ensure a balance between timely treatment of potentially life-threatening infections and early modification and de-escalation of antibiotic regimens where appropriate.^19^ Historically, ASPs have focused mainly on the inpatient setting.^22^ However, it is crucial for these programs to be holistic and extend efforts to the outpatient setting for a broader impact.

Facilities may design outpatient AS strategies to track and report clinician or facility-level data for high-priority conditions such as URTI, bronchitis, and uncomplicated sinusitis.^3^ Prior studies support the implementation of institutional evidence-based treatment guidelines, shorter courses of antibiotics, and early de-escalation to oral agents for conditions such as febrile neutropenia, where appropriate, for successful AS among immunocompromised patients.^20,21,24^ Studies specifically assessing antibiotic prescription practices in oncology clinics remain limited and highlight the high rates of inappropriate prescriptions for conditions such as acute bacterial skin-skin structure infection (ABSSSI), urinary tract infection (UTI), URTI and lower respiratory tract infection (LRTI).^22,25,26^

Existing literature establishes the role of institutional guidelines in improving outpatient antibiotic consumption. Through this study, we examined outpatient antibiotic prescription trends at a cancer center in Pakistan over a 5-year period, spanning from 2018 to 2023, and elucidated the impact of treatment guidelines on antibiotic prescriptions for UTI, URTI, LRTI, and diarrhea.

## Methods

### Study setting and design

We conducted this retrospective, observational study at Shaukat Khanum Memorial Trust (SKMT), Pakistan, a health system comprising two tertiary care cancer centers providing treatment to pediatric and adult cancer patients. Approximately 10,000 to 14,000 cancer patients are registered for treatment per year. A total of 1301 physicians from 38 outpatient specialties provided care to cancer patients during the study period. A program for antibiotic restriction has been in place at SKMT since 1995 but a formal ASP was established in 2010, chaired by an infectious diseases (ID) physician with dedicated ID pharmacists and support from the microbiology and information technology (IT) departments. Broad-spectrum oral antibiotics were restricted and dispensed following ID approval. However, physicians could prescribe unrestricted antibiotics for any indication and the desired duration. Antibiotics were then reviewed by pharmacy for dose adjustment. Antibiotic stewardship efforts at our center initially focused primarily on inpatient antibiotics. The ID team developed institutional guidelines for the management of URTI and diarrhea in July 2022, and skin and skin structure infection (SSSI), UTI, and LRTI in February 2021 (Supplement 1). These were incorporated into the electronic medical record (EMR) for easy access by prescribers. This information was disseminated through an email to physicians across the trust and incorporated into the institutional pharmacy newsletter following the introduction of guidelines into the EMR and annually thereafter. In addition to electronic dissemination, we educated newly hired physicians on these guidelines during orientation. We did not utilize order sets. Additionally, data on guideline access were not tracked. Other outpatient AS activities remained limited.

### Study objectives

The primary objective of the study was to determine the impact of treatment guidelines on “inappropriate” and “unnecessary” outpatient antibiotic prescriptions. The secondary objective was to compare “inappropriate” and “unnecessary” antibiotic prescribing trends for pediatric and adult populations over the defined study period.

### Data collection

The organization uses an Oracle-based (Oracle Corporation, Redwood Shores, CA, USA) home-grown EMR since 2001.^27^ This served as the data source for the study. We determined the total number of outpatient visits and obtained data on the following variables for all cancer patients prescribed oral outpatient antibiotics between July 1^st^ 2018 and July 31^st^ 2023 through a report generated by the IT department: age, sex, underlying malignancy, name, dose and duration of the antibiotic prescribed, date of prescription, and the specialty clinics prescribing the antibiotics. Patients were grouped by age as pediatric if aged <18 years and adult if ≥18 years of age. Fever was defined as documented temperature of ≥ 38°C. Diarrhea was defined as ≥3 loose stools over a 24-hour period. Indications for antibiotic prescription were categorized as fever without any other associated diagnosis, bacteremia, LRTI, URTI, SSSI, UTI, diarrhea, hepatobiliary infection, and bone and joint infection. All other indications were grouped as “other.” Antibiotics prescribed without documentation of the indication were labeled as “prescribed without a clear indication.” All categories were mutually exclusive. We considered antibiotics “unnecessary” if the patients did not meet the criteria for antibiotic prescription based on institutional guidelines (Supplement 1). Antibiotics were considered “inappropriate” if the antibiotic prescription did not conform to institutional guidelines in terms of the indication, antibiotic choice, dose, or duration (Supplement 1).

We identified the top 10 antibiotics prescribed and the 3 leading specialties prescribing antibiotics. Physician notes were reviewed for all patients with an antibiotic prescription. The initial review was performed by first and second-year internal medicine residents to determine the indication for antibiotic prescription. Prophylactic antibiotics prescribed as part of chemotherapy protocols were excluded. Physician notes were then reviewed by a group of third-year internal medicine residents and ID fellows to confirm the indication for antibiotic prescriptions for UTI, URTI, LRTI, and diarrhea and assess if these were guideline-discordant. These data were reviewed by an ID physician to categorize the prescriptions as “inappropriate” or “unnecessary.”

### Statistical analysis

Frequencies and proportions were reported for categorical variables and mean and standard deviation for continuous variables. We calculated the cost of antibiotics prescribed without a clear indication and those considered inappropriate or unnecessary for LRTI, UTI, URTI, and diarrhea in United States Dollars($). We used the two-sample χ^2^ test to compare the proportion of inappropriate and unnecessary antibiotics prescribed for UTI, URTI, LRTI, and diarrhea before and after the incorporation of treatment guidelines into the EMR. A similar analysis was performed to compare the proportion of inappropriate and unnecessary antibiotics prescribed for pediatric and adult patients. All analyses were two-tailed, with a level of significance of 0.05. Statistical analysis was performed using SPSS (IBM SPSS Statistics 27.0.1).

Results: A total of 773,065 outpatient visits occurred during the study period, with 674,686 (87.3%) adult and 98,379 (12.7%) pediatric visits. Antibiotics were prescribed during 50,984 visits. Of these, 27,043 patients received antibiotics for prophylaxis or as part of chemotherapeutic regimens and were excluded. The remaining 23,941 patients receiving antibiotic prescriptions for any other indication or without clear documentation of the indication were included in the study. In this cohort, adults were prescribed antibiotics during 19,436 (2.9%) visits and pediatric patients during 4,505 (4.6%) visits. Patient demographics are summarized in Table 1. The top indication for antibiotic prescription among adults was SSSI (n = 4,455, 22.9%), followed by UTI (n = 2,563; 13.2%), URTI (n = 1,560; 8.0%) and LRTI (n = 1,083; 5.6%). The most common indication for antibiotic prescription among pediatric patients was URTI (n = 1,200; 26.6%), followed by SSSI (n = 704; 15.6%), LRTI (n = 235; 5.2%), and diarrhea (n = 223; 5.0%). Antibiotics were prescribed without a clear indication for 3,828 (19.7%) adult and 920 (20.4%) pediatric patients (Table 1).

**Table 1.** Baseline demographics and indications for antibiotic prescribing among adult and pediatric patients

	Adults (N = 19436)n (%)	Pediatric (N = 4505) n (%)
Age in years (mean+/– STD)	49.3 (+/–14.3)	9.1 (+/– 3.5)
Sex		
Male	9028 (46.4)	2986 (66.3)
Female	10408 (53.6)	1519 (33.7)
Underlying malignancy		
Hematologic	2695 (13.9)	3078 (68.3)
Solid organ	16741 (86.1)	1427 (31.7)
Indications for antibiotic prescription		
SSSI	4455 (22.9)	704 (15.6)
UTI	2563 (13.2)	123 (2.7)
URTI	1560 (8.0)	1200 (26.6)
LRTI	1083 (5.6)	235 (5.2)
Diarrhea	756 (3.9)	223 (5.0)
Fever	280 (1.4)	136 (3.0)
Hepatobiliary infection	128 (0.7)	2 (0.0)
Bacteremia	79 (0.4)	38 (0.8)
Bone and joint infection	68 (0.3)	39 (0.9)
Other	4636 (23.9)	885 (19.6)
Prescribed without a clear indication	3828 (19.7)	920 (20.4)

STD, standard deviation; SSSI, skin and skin structure infection; URTI, upper respiratory tract infection; UTI, urinary tract infection; LRTI, lower respiratory tract infection.

**Figure 1.**
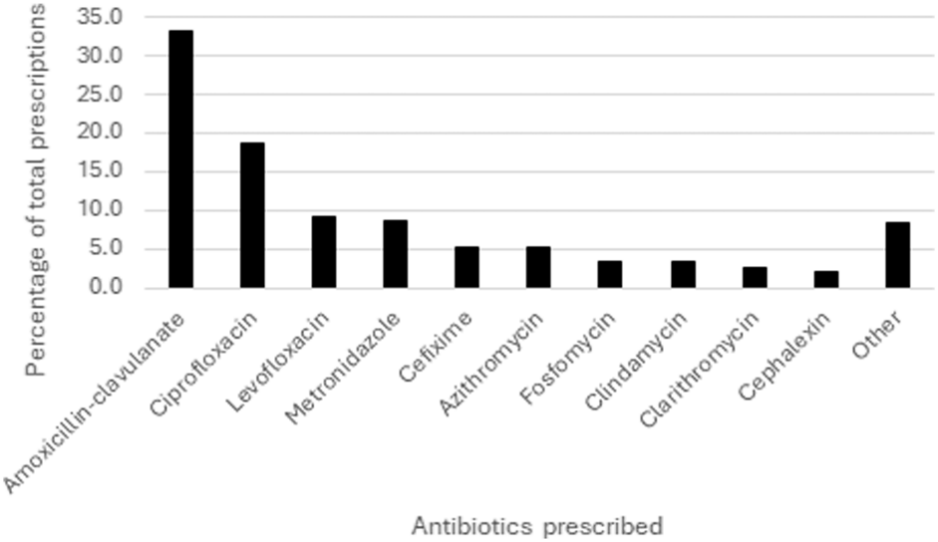
Most frequently prescribed antibiotics in the outpatient setting.

Medical oncology was the top antibiotic-prescribing specialty (n = 4,959; 20.7%), followed by pediatric oncology (n = 4,378; 18.3%) and radiation oncology (n = 3,309; 13.8%). Amoxicillin-clavulanate (n = 7,964; 33.3%), ciprofloxacin (n = 4478; 18.7%) and levofloxacin (n = 2,222; 9.3%) were most frequently prescribed (Figure 1). A large proportion of antibiotic prescriptions for URTI, diarrhea, UTI, and LRTI were either inappropriate (n = 6,695; 86.5%) or unnecessary (n = 5,534; 71.5%) (Table 2). Prescribers most commonly deviated from the appropriate choice of antibiotics for UTI (n = 1,785; 66.5%). Inappropriate antibiotic duration was most commonly observed for patients with LRTI (n = 268; 20.3%) (Table 2).

**Table 2.** Frequency of inappropriate or unnecessary outpatient antibiotics prescribed for LRTI, UTI, diarrhea, and URTI and frequency of antibiotics with incorrect choice, dose, and duration of treatment prescribed for these indications

Antibiotic indication	Inappropriate antibiotic choice n (%)	Incorrect dose n (%)	Inappropriate duration of therapy n (%)	Inappropriate antibiotics^a^ n (%)	Unnecessary antibiotics^a^ n (%)	Appropriate antibiotics n (%)
LRTI (n = 1318)	115 (8.7)	80 (6.1)	268 (20.3)	724 (54.9)	441 (33.5)	594 (45.0)
UTI (n = 2686)	1785 (66.5)	169 (6.3)	80 (3.0)	2357 (87.8)	1717 (63.9)	329 (12.2)
Diarrhea (n = 979)	18 (1.8)	126 (12.9)	125 (12.9)	899 (91.8)	674 (68.8)	80 (8.2)
URTI (n = 2760)	96 (3.5)	198 (7.2)	244 (8.8)	2715 (98.4)	2702 (97.9)	45 (1.6)
Total (n = 7743)	2014 (26.0)	573 (7.4)	717 (9.3)	6695 (86.5)	5534 (71.5)	1048 (13.5)

LRTI, lower respiratory tract infection; UTI, urinary tract infection; URTI, upper respiratory tract infection.

Inappropriate antibiotics: antibiotic prescriptions that did not conform to institutional guidelines in terms of the indication, antibiotic choice, dose, or duration.

Unnecessary antibiotics: antibiotics that did not meet the prescription criteria based on institutional guidelines.

a Inappropriate” and “unnecessary” antibiotics do not represent mutually exclusive categories.

The overall cost of inappropriately prescribed antibiotics was the highest for UTI ($12,973), followed by URTI ($5,218), LRTI ($1,711), and diarrhea ($832), with an expenditure of $8,978 on antibiotics prescribed without a clear indication, amounting to a total of $29,712.

The two-sample χ^2^ test revealed a statistically significant decrease in the proportion of inappropriate antibiotics prescribed for UTI (91.3% vs 84.0%; *P* ≤ .001) and diarrhea (92.6% vs 87.0%; *P* = .031) and unnecessary antibiotics for diarrhea (90.2% vs 83.2%; *P* = .016) following the introduction of treatment guidelines into the EMR (Table 3). We observed a higher proportion of unnecessary prescriptions for LRTI (41.7% vs 31.7%; *P* = 0.003) and inappropriate antibiotics for UTI (95.1% vs 87.4%; *P* = .011) for pediatric patients when compared to adult patients but no significant difference for URTI and diarrhea (Supplement 2).

**Table 3.** Two-sample χ^2^ test comparing the proportion of inappropriate and unnecessary antibiotic prescriptions before and after the introduction of treatment guidelines in the EMR

Antibiotic indication (date of guideline incorporation in the EMR)	Category of antibiotic prescription	Prescriptions before guideline incorporation in the EMR n/N (%)	Prescriptions after guideline incorporation in the EMR n/N (%)	*P* value
LRTI (February 2021)	Inappropriate	442/797 (55.5)	282/521 (54.1)	.635
	Unnecessary	251/797 (31.5)	190/521 (36.5)	.061
UTI (February 2021)	Inappropriate	1263/1383 (91.3)	1094/1303 (84.0)	<.001
	Unnecessary	890/1383 (64.4)	827/1303 (63.5)	.634
URTI (July 2022)	Inappropriate	2331/2371 (98.3)	384/389 (98.7)	.562
	Unnecessary	2320/2371 (97.8)	382/389 (98.2)	.654
Diarrhea (July 2022)	Inappropriate	785/848 (92.6)	114/131 (87.0)	.031
	Unnecessary	765/848 (90.2)	109/131 (83.2)	.016

EMR, electronic medical record; LRTI, lower respiratory tract infection; UTI, urinary tract infection; URTI, upper respiratory tract infection.

Inappropriate antibiotics: antibiotic prescriptions that did not conform to institutional guidelines in terms of the indication, antibiotic choice, dose, or duration.

Unnecessary antibiotics: antibiotics that did not meet the prescription criteria based on institutional guidelines.

## Discussion

To date, a limited number of oncology-focused studies provide an in-depth analysis of outpatient antibiotic prescription practices for pediatric and adult cancer patients. The results of our study revealed a high proportion of inappropriate or unnecessary antibiotic prescriptions for URTI, LRTI, diarrhea, and UTI despite a low overall prescription rate at our institution. Notably, only 2.9% of adult and 4.6% of pediatric visits resulted in antibiotic prescriptions over the study period. Furthermore, we found no associated diagnosis of infection for about 20% of the antibiotics prescribed for patients from both age groups. Treatment guideline incorporation into the EMR was associated with a decline in inappropriate or unnecessary outpatient antibiotic prescriptions for diarrhea and inappropriate antibiotics for UTI. Inappropriate and unnecessary antibiotics or those prescribed without a clear indication accounted for a direct total healthcare expenditure of $29,712.

Outpatient antibiotic prescribing varies geographically. In the USA, antibiotics are prescribed following 10% of adult and 20% of pediatric outpatient appointments.^3^ A study from India reported antibiotic prescriptions following 17.5% of outpatient encounters.^5^ Outpatient antibiotic prescription rates vary from 12% at tertiary centers to 81% at primary healthcare facilities across Pakistan.^10,12^ The comparatively lower outpatient antibiotic prescription rates at our institution are unexpected but encouraging as cancer patients are often over treated for confirmed or suspected infection.^20,21^ This may be attributed to a spillover effect from a robust inpatient ASP as physicians at our institution provide care in the inpatient and outpatient setting, using a common EMR. The annual national expenditure on inappropriate antibiotics among insured adults in the USA is the highest for pharyngitis ($49.6 million), followed by sinusitis ($19.1 million) and viral URTI ($2.7 million).^14^ These figures cannot be directly compared to our patient population owing to the varied access to resources and stark contrast in the healthcare delivery models in the two countries.

Prescriptions for common indications such as URTI, LRTI, and SSSI significantly contribute to the burden of inappropriate and unnecessary outpatient antibiotics. One-third of adult cancer patients may be prescribed antibiotics for URTI, despite identification of viral etiologies among up to 75% of the patients.^26^ In a study conducted in outpatient oncology clinics, Chew *et al.* noted a high proportion of inappropriate antibiotics for SSSI (24.1%), URTI (15.9%), and LRTI (22.4%).^22^ Similarly, in a retrospective cohort study assessing outpatient antibiotic prescriptions among adult cancer patients, 64% received inappropriate antibiotics, with the highest proportion of inappropriate prescriptions for LRTI (85%), followed by URTI (73%), UTI (65%) and ABSSSI (48%). The most common parameters associated with suboptimal prescriptions were antibiotic dose (54%), choice (53%), and treatment duration (23%).^25^ Studies evaluating antibiotic prescription trends among pediatric oncology patients identified incorrect dose and guideline-discordant antibiotic selection as the most common areas for pharmacy intervention.^19,24^ Several studies centered on non-cancer patients reported similar trends with inappropriate or unnecessary antibiotics prescribed for conditions such as sore throat, cough, rhinorrhea, bronchiolitis, and gastroenteritis or without an associated diagnosis code of infection.^28–31^ According to an estimate, about 75% of adult patients may be prescribed longer than the recommended duration of antibiotics in the outpatient setting.^6^ The most common indications for antibiotics at our institution were similar to those at other centers. Additionally, we identified diarrhea among the top indications for antibiotic prescription. This is expected as cancer patients commonly experience diarrhea as a side effect of oncologic treatments and acute diarrheal illnesses are highly prevalent in our community.^32,33^ The overall proportion of inappropriate (n = 6,695; 86.5%) or unnecessary (n = 5,534; 71.5%) antibiotics was alarmingly high for URTI, diarrhea, UTI, and LRTI. Among prescription parameters, deviation from the recommended antibiotic choice (n = 2,014; 26.0%) most commonly contributed to inappropriate prescription. Despite differences in the predominant malignancy type among adults (solid organ; n = 16741; 86.1%) and children (hematologic; n = 3078; 68.3%), higher rates of inappropriate or unnecessary prescriptions among pediatric patients were limited to two categories: unnecessary prescriptions for LRTI (31.7% vs 41.7%; *P* = .003) and inappropriate prescriptions for UTI (87.4% vs 95.1%; *P* = .011) for adults compared to pediatric patients. Our results demonstrate that physicians at our center based prescribing on the underlying indication, irrespective of the underlying malignancy.

Access and adherence to treatment guidelines are crucial to improve the use of antibiotics.^16^ In an interrupted time series analysis, Lim *et al.* noted a decrease in antibiotic consumption and 54.6% improvement in appropriate prescriptions (*P* < .0001) with the introduction of treatment guidelines in Malaysian primary care clinics.^34^ In another study, antibiotic consumption decreased by 4% over a 10-year period following the introduction of treatment guidelines for general practitioners.^28^ In a recent systematic review, Oliveira et al noted higher compliance with treatment guidelines for respiratory tract infections among pediatric prescribers compared to providers treating adult patients.^16^ We noted a significant decline in the proportion of inappropriate antibiotic prescriptions for UTI and diarrhea and unnecessary prescriptions for diarrhea with the introduction of institutional treatment guidelines. The impact of guidelines on prescriptions for URTI and LRTI at our center may have been dampened by the coronavirus disease 2019 (COVID-19) pandemic which occurred during the same time period. Similar findings have been reported in other studies. According to an estimate, the overall use of azithromycin surged, closely following the peaks in COVID-19 cases in the USA.^15^ In another study conducted in England, while the proportion of antibiotic prescriptions for LRTI decreased by 20%, those for URTI increased by 15.9%, and over 60% were dispensed without an associated diagnosis of infection during the COVID-19 pandemic.^35^

Our study has several limitations. We conducted this retrospective study at a single institution. Our results heavily rely upon physician documentation in EMR. We performed a before-and-after analysis rather than an interrupted time-series analysis to compare antibiotic prescribing trends. This may have biased our results. We did not assess antibiotic-related adverse events, the incidence of subsequent infections with MDROs among our patients or determine the indirect healthcare costs associated with antibiotic misuse. Furthermore, our results may not be generalizable to the general population, to patients treated at non-private cancer centers or at institutions without ASPs.

This is among the first studies assessing the impact of treatment guidelines on outpatient antibiotics for cancer patients and comparing prescription trends between pediatric and adult patients, spanning over a duration of 5 years. Our data collection involved a rigorous three-step review of patient records. Additionally, the use of EMR allowed us to accurately capture prescription data from all healthcare providers.

Our results support targeting URTI, diarrhea, UTI, and LRTI as high-priority conditions for outpatient oncology-focused AS interventions. Our findings highlight the gaps in our outpatient ASP and underscore the need for strategies such as prescriber education on treatment guidelines and mandatory documentation of clinical indication while prescribing antibiotics. Further studies with a multi-center, prospective design and the use of methodologies such as an interrupted time series analysis could further enhance the assessment of outpatient antibiotic trends among cancer patients.

## Supporting information

Abbas et al. supplementary material 1 Abbas et al. supplementary material

Abbas et al. supplementary material 2 Abbas et al. supplementary material

## Supplementary material

The supplementary material for this article can be found at https://doi.org/10.1017/ash.2025.2
