# Supplementary material for: Examining the impact of treatment guidelines on outpatient antibiotic prescription trends at a cancer center in Pakistan
Source: Antimicrob Steward Healthc Epidemiol. 2025 Feb 20;5(1):e51. doi: 10.1017/ash.2025.2 (PMC11869060; doi:10.1017/ash.2025.2)
Supplement: Abbas et al. supplementary material 1 — Abbas et al. supplementary material [file S2732494X25000026sup001.pdf]

Disclaimer: These guidelines were prepared by the department of Internal Medicine, Shaukat Khanum Memorial Cancer Hospital and Research Center in conjunction with the pharmacy department. They are intended to serve as a general guide based on available medical literature at the time of development and are not intended to replace clinical judgement or dictate care of individual patients.

Treatment Guidelines for Pneumonia

Community-acquired Pneumonia:

Definition: Acute infection of the pulmonary parenchyma, accompanied by an acute infiltrate consistent with pneumonia on chest radiograph or auscultatory findings, acquired in the community. Patients must not have been hospitalized recently, nor had regular exposure to the health care system

Common Bacteria involved Community acquired infection:

Bacteria: *Streptococcus pneumonia, Haemophilus influenzae, Mycoplasma pneumonia, Legionella pneumophila, Staphylococcus aureus, Moraxella catarrhalis, Anaerobes, Gram-negative bacilli (e.g., Klebsiella pneumonia)*

CURB-65 Score:

Hospitalization should be based on the severity-of-illness scores:

a) Confusion, new onset
b) Urea greater than 19 mg/dL
c) Respiratory rate of 30 breaths/minute or more
d) Blood pressure of 90 mm Hg or less systolic or diastolic of 60 mm Hg or less
e) Age 65 years or older

CURB 65		
Score	Risk of Death at 30 Days (%)	Location of Therapy
0	0.7	Outpatient
1	2.1	Outpatient
2	9.2	Outpatient or inpatient
3	14.5	Inpatient (± ICU)
4	40	Inpatient (± ICU)
5	57	Inpatient (± ICU)

A) CAP empiric treatment, outpatient (low risk, with or without co-morbidities):

1) Amoxicillin-clavulanic Acid 1g q12h for 7 days (If suspicion of atypical pneumonia, add azithromycin 500mg on day 1, then 250mg q24h on remaining days for 4 days or doxycycline100mg bid 5-7 days, if suspicion of atypical pneumonia)
2) Levofloxacin 750mg q24h for 5-7 days (Alternative option in case of penicillin allergy, diabetic or immunocompromised)

Disclaimer: These guidelines were prepared by the department of Internal Medicine, Shaukat Khanum Memorial Cancer Hospital and Research Center in conjunction with the pharmacy department. They are intended to serve as a general guide based on available medical literature at the time of development and are not intended to replace clinical judgement or dictate care of individual patients.

B) CAP empiric treatment, inpatient, non-severe (non-ICU, without Risk Factors for MRSA and P. aeruginosa):

1) A fluoroquinolone with significant Streptococcus pneumoniae activity (PO Levofloxacin 750 q24)
2) An intravenous non-pseudomonal β-lactam (e.g., ceftriaxone 2g q24h, cefotaxime 2g q8h) + a macrolide such as azithromycin (500mg q24h 5 days) doxycycline 100mg bid may be used as an alternative to azithromycin).

Total duration of therapy 7 days, (If patient fails to respond, consider ID review)

C) In the Inpatient Setting, Adults with CAP and Risk Factors for MRSA or *P. aeruginosa* (Those patients who have any one of several potential risk factors for antibiotic-resistant pathogens, , hospitalization for >2 days in the last 90 days, receipt of home infusion therapy, chronic dialysis, home wound care, or a family member with a known antibiotic-resistant pathogen, immunocompromised)

1) Piperacillin/Tazobactam 4.5g q6h (I/V) + Macrolide (PO preferred over IV) + Vancomycin (I/V) (loading dose 25-30mg/kg I/V, followed 15mg/kg q12 I/V, target trough levels 15-20mcg/ml, draw serum sample 30-60 min before 4th dose)
2) In case of penicillin allergy , Piperacillin/Tazobactam can be replaced with Meropenem 1g q8h or Imipenem/Cilastatin 500mg q6h or 1g q8h.

Follow culture sensitivity results for optimization of antibiotics.
Total duration of therapy 7 days, (If the patient fails to respond, consider ID review)

Hospital Acquired Pneumonia

Pneumonia that develops 48 hours post admission

1) Piperacillin/Tazobactam 4.5g q6h (I/V) + Vancomycin (I/V) (loading dose 25-30mg/kg I/V, followed 15mg/kg q12 I/V, target trough levels 15-20mcg/ml, draw serum sample 30-60 min before 4th dose)
2) In case of penicillin allergy , Piperacillin/Tazobactam can be replaced with Meropenem 1g q8h or Imipenem/Cilastatin 500mg q6h or 1g q8h)

Follow culture sensitivity results for optimization of antibiotics.

Disclaimer: These guidelines were prepared by the department of Internal Medicine, Shaukat Khanum Memorial Cancer Hospital and Research Center in conjunction with the pharmacy department. They are intended to serve as a general guide based on available medical literature at the time of development and are not intended to replace clinical judgement or dictate care of individual patients.

Total duration of therapy is 7 days (If the patient fails to respond, consider ID review).

Doses In pediatrics:

**Antibiotic**	**Dose**	**Duration of therapy**
Levofloxacin	PO/IV 16-20mg/kg/day (max dose 750mg/day) in two divided doses	7 days
Piperacillin/Tazobactam	90mg/kg/dose q6h	7 days
Meropenem	20mg/kg/dose q8h	7 days
Imipenem/Cilastatin	60-100mg/kg/day in 4 divided doses	7 days
Azithromycin	PO/IV 15mg/kg/dose	5 days
Co-amoxiclave	PO 45-90mg/kg/ day amoxicillin in two divided doses	7 days

Total duration of therapy 7 days, (If the patient fails to respond, consider ID review)

Ref: Domachowske, J., & Suryadevara, M. (2020). Community-Acquired Pneumonia. In *Clinical Infectious Diseases Study Guide* (pp. 53-59). Springer, Cham.
(Modified based on hospital and community antibiogram)
American Journal of Respiratory and Critical Care Medicine, Volume 200, Issue 7, 1 October 2019, Pages e45-e67
Published: 01 October 2019

Disclaimer: These guidelines were prepared by the department of Internal Medicine, Shaukat Khanum Memorial Cancer Hospital and Research Center in conjunction with the pharmacy department. They are intended to serve as a general guide based on available medical literature at the time of development and are not intended to replace clinical judgement or dictate care of individual patients.

Treatment Guidelines for Diarrhea

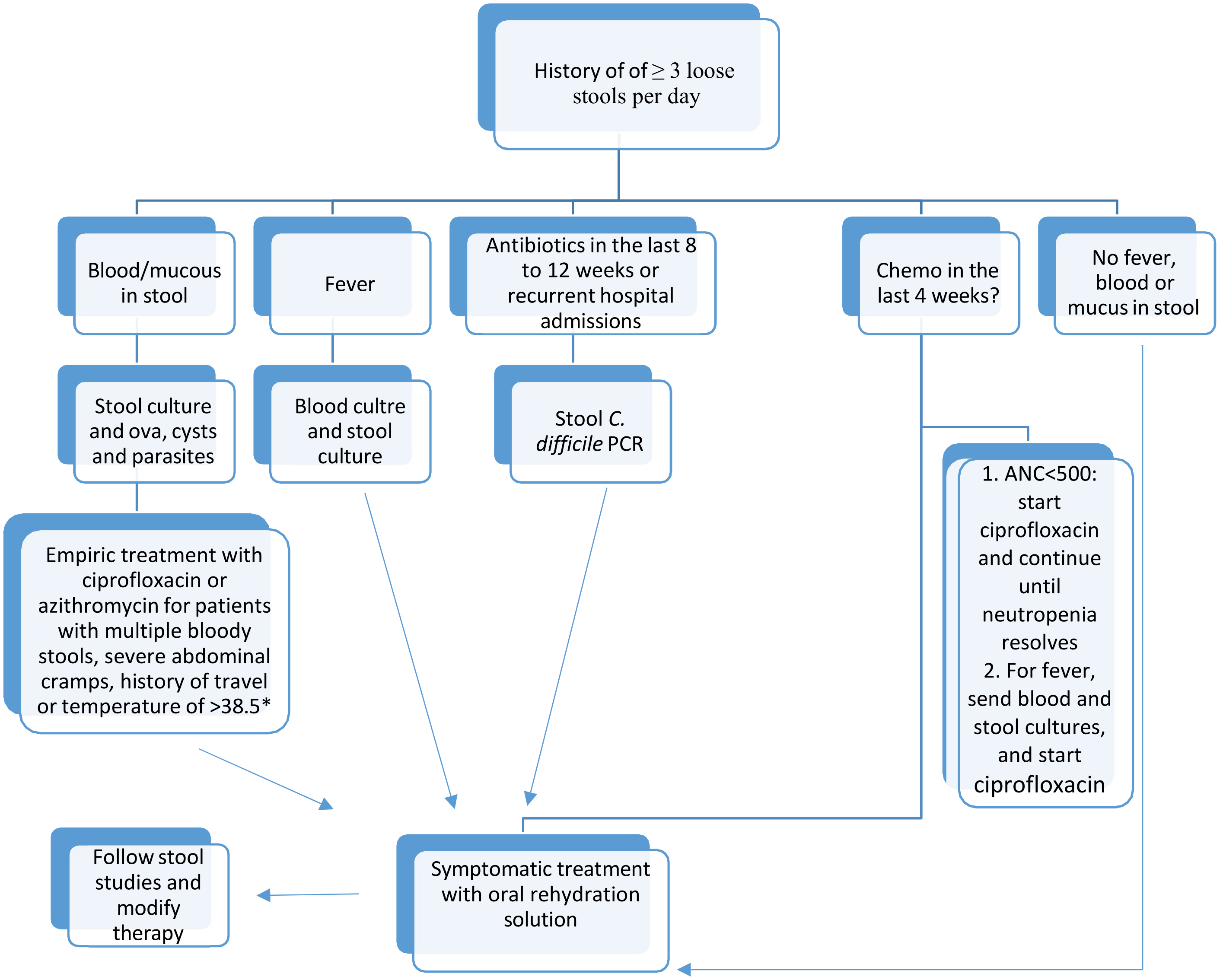

For patients with hemodynamic instability, refer to EAR for IV fluids. In case of fever and hypotension, send septic work up including blood and stool cultures and start piperacillin-tazobactam empirically.

Disclaimer: These guidelines were prepared by the department of Internal Medicine, Shaukat Khanum Memorial Cancer Hospital and Research Center in conjunction with the pharmacy department. They are intended to serve as a general guide based on available medical literature at the time of development and are not intended to replace clinical judgement or dictate care of individual patients.

If the patient improves and becomes stable discharge with oral Ciprofloxacin 500mg orally twice daily with early follow up. For persistent hemodynamic instability, admit the patient.

*Dose of ciprofloxacin: 500 mg orally twice daily
(Pediatrics: 10 mg/kg/dose twice daily; maximum dose: 500 mg/dose)
Dose of azithromycin 500 mg orally daily
(Pediatrics: Oral: 10 mg/kg/dose ; maximum dose: 500 mg/dose)

Reference : IDSA guideline for infectious diarrhea/Mendel and Douglas, chapter 96; ASCO guidelines for post chemotherapy diarrhea

Disclaimer: These guidelines were prepared by the department of Internal Medicine, Shaukat Khanum Memorial Cancer Hospital and Research Center in conjunction with the pharmacy department. They are intended to serve as a general guide based on available medical literature at the time of development and are not intended to replace clinical judgement or dictate care of individual patients.

Treatment Guidelines for Upper Respiratory Tract Infection

Symptoms concerning for an upper respiratory tract infection (e.g. fever, cough, throat ache)

↓

- **Fever over 38°C**
- **Tender anterior cervical lymphadenopathy or lymphadenitis**
- **Tonsillar exudate**
- **Absence of cough**

(1 point for each)

↓

Score of 3 or 4 and positive throat swab culture[1]

↓

Likely bacterial infection
Treat with antibiotics. One of the following may be prescribed:
Co-amoxiclav 1g orally twice daily for 5 days treatment(Pediatric: 20 to 40 mg amoxicillin/kg/day orally in divided doses 3 times daily; maximum daily dose: 1,500 mg/day)

Or cefixime 400 mg orally once daily for 5 days
(Pediatrics: 8 mg/kg/day orally once daily or in divided doses every 12 hours ; maximum daily dose: 400 mg/day)

If allergic to penicillin, may treat with Clarithromycin 250 mg orally twice daily for 5 days(Pediatrics: 7.5 mg/kg/dose every 12 hours for 10 days; maximum dose: 250 mg/dose)

Score of 0, 1 or 2

↓

Rule out Viral causes
Supportive

Disclaimer: These guidelines were prepared by the department of Internal Medicine, Shaukat Khanum Memorial Cancer Hospital and Research Center in conjunction with the pharmacy department. They are intended to serve asa general guide based on available medical literature at the time of

Suspect bacterial sinusitis with more than 10 days of symptoms such as discolored/purulent nasal discharge, severe localized unilateral pain particularly over the teeth, jaw or sinuses, fever and marked deterioration after an initial milderphase of illness. One of the following may be prescribed:

Co-amoxiclav 625g orally thrice daily for 7-14 days*Or cefixime 400 mg orally once daily for 7-14 days*

If allergic to penicillin, may treat with Clarithromycin 250 mg orally twice daily for 7-14 days depending upon clinicalresponse*

(*Pediatric dosing as above)

[1]Swabbing the throat and testing for Group A Streptococcal (GAS) pharyngitis by culture should be performed because the clinical features alone do not reliably discriminate between GAS and viral pharyngitis except when overt viral features like rhinorrhea, cough, oral ulcers, and/or hoarseness are present.

Reference - Kim, Nee Na; Marikar, Dilshad (2019). Antibiotic prescribing for upper respiratory tract infections: NICE guidelines. Archives of disease in childhood - Education & practice edition, (), edpract-2018-316159–. doi:10.1136/archdischild-2018-316159

Clinical Practice Guideline for the Diagnosis and Management of Group A Streptococcal Pharyngitis: 2012 Update by the Infectious Diseases Society of America

Disclaimer: These guidelines were prepared by the department of Internal Medicine, Shaukat Khanum Memorial
Cancer Hospital and Research Center in conjunction with the pharmacy department. They are intended to serve asa general guide based on available medical literature at the time of

Treatment Guidelines for Urinary tract Infections

Introduction

**Category**	**Definition**
Uncomplicated UTI	-Lower urinary symptoms (dysuria, frequency, and urgency) in otherwise healthy non-pregnant women
Complicated UTI	-Pregnant women, men, obstruction, immunosuppression, renal failure, renal transplantation, urinary retention from neurologic disease, and individuals with risk factors that predispose to persistent or relapsing infection (e.g., calculi, indwelling catheters or other drainage devices)<br>-Health care associated
Asymptomatic bacteriuria	-Women: Two consecutive voided urine specimens with isolation of the same bacteria at ≥ 10^5 CFU/mL<br><br>-A single catheterized urine specimen with 1 bacteria isolated ≥ 10^2 CFU/mL

Most common Pathogens

**Type**	**Common Uropathogens**
Uncomplicated UTI	*Escherichia coli (E.coli) , Enterococcus spp., Klebsiella pneumonia, Proteus mirabilis*
Complicated UTI	(Similar to uncomplicated UTI Antibiotic-resistant)<br>*Escherichia coli, Pseudomonas aeruginosa, Acinetobacter baumannii, Enterococcus spp., Staphylococcus spp.*
Recurrent UTI	*Proteus mirabilis, Klebsiella pneumonia, Enterobacter spp.,* Resistant *Escherichia coli, Enterococcus spp., Staphylococcus spp.,*

Empiric Antibiotic Recommendations According to Type of UTIs (and our hospital Anti-biogram for E.coli, follow culture sensitivities for definite therapy)

**Antibiotics**	**Dose**	**Duration**	**comments**
**Acute Uncomplicated UTI**			
Nitrofurantoin (Furadantin)	100mg PO Q6h	7days	Avoid during first trimester or near term, Avoid if CrCl<50ml/min

Disclaimer: These guidelines were prepared by the department of Internal Medicine, Shaukat Khanum Memorial
Cancer Hospital and Research Center in conjunction with the pharmacy department. They are intended to serve asa general guide based on available medical literature at the time of

Nitrofurantoin (Macrobid)	100mg PO q12h	5 days	Avoid if CrCl<50ml/min
Fosfomycin (Sachet)	3g PO	One dose	No such renal dose adjustment
Fosfomycin Capsule	500mg PO q8h	5 days	No such renal dose adjustment
**Acute Uncomplicated Pyelonephritis,<br>Acute Complicated Cystitis or CA-UTI without upper tract symptoms**			
Piperacillin-tazobactam	4.5g IV q6hr	7 days	Renal dose adjustment needed
Meropenem	1g IV q8hr	7 days	Use If reports of allergy to penicillin, Renal dose adjustment
**Acute Complicated Pyelonephritis or Urosepsis or CA-UTI patients who are severely ill (Empiric antibiotics should be switched to definite one according to culture sensitivities, remove catheter)**			
Piperacillin/tazobactam	4.56 IV q6h	7 days	Renal dose adjustment needed
Meropenem	1g IV q8h	7 days	Use If reports of allergy to penicillin, Renal dose adjustment
**Asymptomatic Bacteriuria in Pregnant Women**			
Nitrofurantoin (Furadantin)	100mg PO Q6h	7days	Avoid during first trimester or near term, Avoid if CrCl<50ml/min
Nitrofurantoin (Macrobid)	100mg PO 12h	5days	Avoid during first trimester or near term, Avoid if CrCl<50ml/min
Fosfomycin Sachet	3g PO		
Co-trimoxazole	960mg PO q12h	3 days	Avoid during first trimester or near term
**UTIs in Pregnant Women**			
Nitrofurantoin (Furadantin)	100mg PO Q6h	7days	Avoid during first trimester or near term, Avoid if CrCl<50ml/min
Nitrofurantoin (Macrobid)	100mg PO q12h	7 days	Avoid during first trimester or near term, Avoid if CrCl<50ml/min
Fosfomycin (Sachet)	3g q72h PO	7 days	
Co-trimoxazole	960 PO q12h	7 days	Avoid during first trimester or near term
**Prevention of Recurrent UTIs** (consult ID consultant for review of such cases)			

Pediatric dosing:

Drug	dose

Disclaimer: These guidelines were prepared by the department of Internal Medicine, Shaukat Khanum Memorial
Cancer Hospital and Research Center in conjunction with the pharmacy department. They are intended to serve asa general guide based on available medical literature at the time of

Nitrofurantion	7mg/kg/day q6h (maximum dose: 100 mg/dose)
Fosfomycin sachet	Children <12 years: Oral: 2g as a single dose<br><br>Children ≥12 years and Adolescents: Oral: 3g as a single dose<br><br>(q72h if needed prolong duration)
Fosfomycin (Suspension)	New born: Oral: 125mg q6h<br>Infant: Oral: 250mg q6h<br>Children: Oral: 500 q8h
Co-trimoxazole (Syrup/tablet)	2 to 24 months: Oral: 6 to 12 mg TMP/kg/day in divided doses every 12 hours<br>>24 months and Adolescents: Oral: 8 mg TMP/kg/day in divided doses every 12 hours for 3 days; longer duration may be required in some patients; maximum single dose: 160 mg TMP
Piperacillin/tazobactam (injection)	IV: 90mg/kg/dose q6 hourly
Meropenem (Injection)	IV: 20mg/kg/dose q8h

Duration of antibiotics: 7 days or at least 3 days after obtaining sterile urine

Monitor renal functions and liver functions while patient is on antibiotics. Consult ID team incase of any confusion or complication.

Follow urine sampling techniques as per recommendations, so that to ensure truerepresentative sample.

Optimize based on culture sensitivities

** (Adjust dose as per

CrCl)

Ref:

1) Lexicomp 2023
2) International clinical practice guidelines for the treatment of acute uncomplicated cystitis and pyelonephritis in women: A 2010 update by the Infectious Diseases Society of America and the European Society for Microbiology and Infectious Diseases.
