# Supplementary material for: Examining the impact of treatment guidelines on outpatient antibiotic prescription trends at a cancer center in Pakistan
Source: Antimicrob Steward Healthc Epidemiol. 2025 Feb 20;5(1):e51. doi: 10.1017/ash.2025.2 (PMC11869060; doi:10.1017/ash.2025.2)
Supplement: Abbas et al. supplementary material 2 — Abbas et al. supplementary material [file S2732494X25000026sup002.pdf]

Supplement 2: Two-sample chi-square test comparing the proportion of inappropriate and unnecessary antibiotic prescriptions between adult and pediatric patients

Antibiotic Indication	Category of antibiotic prescription	Prescriptions for adult patients n/N (%)	Prescriptions for pediatric patients n/N (%)	P value
LRTI	Inappropriate	591/1083 (54.6)	133/235 (56.6)	0.572
	Unnecessary	343/1083 (31.7)	98/235 (41.7)	0.003
UTI	Inappropriate	2240/2563 (87.4)	117/123 (95.1)	0.011
	Unnecessary	1633/2563 (63.7)	84/123 (68.3)	0.302
URTI	Inappropriate	1536/1560 (98.5)	1179/1200 (98.3)	0.664
	Unnecessary	1530/1560 (98.1)	1172/1200 (97.7)	0.456
Diarrhea	Inappropriate	701/756 (92.7)	198/223 (88.8)	0.059
	Unnecessary	680/756 (89.9)	194/223 (87.0)	0.211

LRTI: lower respiratory tract infection; UTI: urinary tract infection; URTI: upper respiratory tract infection
